# Efficacy of treadmill exercises on arterial blood oxygenation, oxygen consumption and walking distance in healthy elderly people: a controlled trial

**DOI:** 10.1186/s12877-016-0283-5

**Published:** 2016-05-25

**Authors:** Ashraf Adel Fahmy Bichay, Juan M. Ramírez, Víctor M. Núñez, Carolina Lancho, María S. Poblador, José L. Lancho

**Affiliations:** Physical & Rehabilitation Medicine, Doctor of philosophy in Health Sciences Methodology & Research with international Doctorate Mention, University of Cordoba, Córdoba, Spain; Department of Morphological Sciences, School of Medicine, University of Córdoba, Avenida de Menéndez Pidal s/n 14071, Córdoba, Spain; Rehabilitation Service, University Hospital Donostia, Donostia, Spain

**Keywords:** Elderly, Aerobic exercise, Cardiovascular system

## Abstract

**Background:**

Regular physical exercise and healthy lifestyle can improve aerobic power of the elderly, although lung capacity gradually deteriorates with age. The aims of the study are: a) to evaluate the therapeutic effect of a treadmill exercise program on arterial blood oxygenation (SaO_2_), maximum oxygen consumption (VO_2max_) and maximum walking distance (MWD) in healthy elderly people; b) to examine the outcome of the program at a supervised short-term and at an unsupervised long-term.

**Methods:**

A prospective, not-randomized controlled intervention trial (NRCT) was conducted. Eighty participants were allocated into two homogeneous groups (training group, TG, *n* = 40; control group, CG, *n* = 40). Each group consisted of 20 men and 20 women. Pre-intervention measures of SaO_2_, VO_2max_ and MWD were taken of each participant 1-week before the training program to establish the baseline. Also, during the training program, the participants were followed up at the 12, 30 and 48th week. The exercise program consisted of walking on a treadmill with fixed 0 % grade of inclination 3 times weekly for 48 weeks; the first 12 weeks were supervised and the remaining 36 weeks of the program were unsupervised. Participants in the control group were encouraged to walk twice a week during 45 min, and received standard recommendations for proper health.

**Results:**

Related to the baseline, the SaO_2,_ VO_2max_, and MWD is greater in the intervention group at the 12^th^ (*p* <.001), 30^th^ (*p* <.001) and 48^th^ week (*p* <.001). Compared with the control group, there was also a significant improvement of SaO_2,_ VO_2max_, and MWD valuesin the intervention group (*p* <.001) at the 12^th^ (*p* <.001), 30^th^ (*p* <.001) and 48^th^ week (*p* <.001). Supervised intervention shows greater improvement of SaO_2,_ VO_2max_, and MWD values than in the unsupervised one.

**Conclusion:**

These results show that performing moderate exercise, specifically walking 3 days a week, is highly recommended for healthy older people, improving aerobic power.

**Trial registration:**

Current Controlled Trials ISRCTN12621097.

## Background

Although the risk of disease and disability increases with age, poor health is not an inevitable result of aging. A healthy lifestyle, which can be achieved by practicing regular physical activity, healthy diet and early detection of diseases, could slow the effects of age [1]. As long as the elderly population continues to increase, it will be essential for family physicians to keep in mind that sedentary patients have to practice physical exercises regularly [2].

Lung function deteriorates gradually with age [3]. A trend towards lower arterial oxygen saturation (SaO_2_) in the elderly suggests that the biological processes of aging in one or more systems are occurring [4]. Maximal oxygen consumption (VO_2max_) is considered as the criterion for measurement of the cardio-respiratory fitness [5]. Some important factors can have an influence on VO_2max_ (e.g.: Age, sex, heredity, body composition, training status, mode of exercise and a number of diseases) [6]. Also aging is associated with a decrease in postural control, gait speed, stride length, the distance walked during the synchronized walking test and there is also an increase in the variability of the gait [7].

Deterioration of lung function could be linked to respiratory impairment, particularly developed as a consequence of smoking or airway infections [8, 9]. In the clinical practice, before attributing any pathological process as related to the decreased lung capacity, aging deterioration must be considered to explain the physiological decline [10].

Many studies have investigated the effect of training on elderly patients suffering from different types of chronic diseases [11–14] or the effect of rehabilitation on elderly subjects after the sequel of certain diseases [15–17], but few studies investigated the effect of exercise on healthy elderly people [18–20].

The cardio-pulmonary exercise test, and the distance walked during the walking tests, has been noted to be useful in the individual prescription of pulmonary rehabilitation and oxygen supplementation [21]. The targeted heart rate for moderate intensity exercise may be considered to be between 40 and 60 % of heart rate reserve, as determined by the exercise test. This heart rate range can be used for the initial prescription of many types of dynamic exercise and can be increased to 85 % (high intensity) if tolerated [22].

Regular exercise provides great health benefits in older people, as well as in the case of younger adults. Despite this, over 75% of older adults are insufficiently active to take advantage of these health benefits [2]. Supervised exercise therapy has shown clinically and statistically significant differences in the improvement of maximal treadmill walking distance, compared to non-supervised exercise therapy regimens [23], and results are usually closely related to adherence to the program [24].

Some studies have investigated the effect of adherence to the exercise program [25, 26], but few of them have studied the degree of compliance of the experimental group, and the impact on the patient [27, 28]. Also, another point of interest is how long the sustained effect of an exercise program lasts.

The aims of this study are: a) to evaluate the therapeutic effect of a treadmill exercise program on arterial blood oxygenation (SaO_2_), maximum oxygen consumption (VO_2max_) and maximum walking distance (MWD) in healthy elderly people controlling the different factors that can influence the results; b) to examine the outcome of the program at a supervised short-term and at an unsupervised long-term. These issues are targeted to report empirical evidence of the importance of maintaining an active lifestyle to slow down the aging effects on health, which, therefore, will lead to greater quality of life in older people.

## Methods

### Participants

**Fig. 1:**
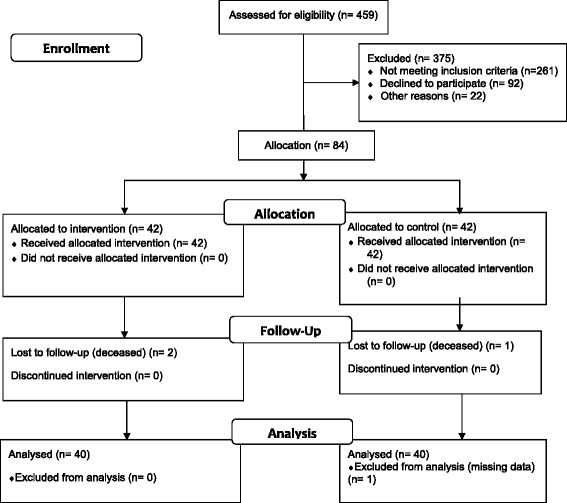
Flow Diagram (according to Consort 2010 Flow Diagram model)

Eighty healthy elderly people, from 459 assessed for eligibility (see Flow Diagram, Fig. 1), were evaluated from the outpatient clinic of ENT & Ophthalmology in a General Hospital in Cairo, Egypt. Previously, 375 were excluded (261 not meeting inclusion criteria, 92 declined to participate, and 22 by others reasons). Eighty four patients were selected for allocation from October 2010 to January 2011, of which two in the intervention group and one in the control group were lost to follow-up. Additionally, one person in the control group was excluded due to missing data. During the conduct of the trial, participants were blinded to the treatment until the study ended. Inclusion criteria to participate in the study were as following; age varying between 60 and 70 years, non-smokers or former smokers for more than 5 years and good general health (without neuromuscular, orthopaedic, neurological or cardiopulmonary conditions). Therefore, exclusion criteria were neuromuscular, orthopaedic, neurological or cardio-pulmonary diseases. Also, we excluded patients with any chronic deficit that would prevent exercise, according to the protocol of Fletcher et al. [22]. After performing a complete medical history, participants were instructed about the procedure and signed an informed consent.

Chest X-rays and ECG were performed to exclude any underlying pathology before the intervention. A 12 Channels Electrocardiogram Biocare (ECG-8080) was utilized to evaluate ECG. Necessary anthropometric measures were recorded such as height and weight to calculate Body Mass Index (BMI) and were introduced into the software along with the demographic data of the participants (name, age and sex). Height and weight scale IPR-scale08 model was used for the measurement of height, weight and BMI calculation. At the same time, instructions were given to the subjects to avoid food intake for 2 h before the test, the measures taken for hygiene and comfort in clothing as well as in footwear and to avoid any unusual physical efforts at least 12 h before the test.

Adherence, defined as the completion of the protocol by the end of it, was 100 %.

### Experimental protocol and outcomes measures

In order to test the effect of a treadmill exercise program on health outcomes of healthy elderly people, a prospective, not-randomized single-blinded controlled intervention trial (NRCT) was conducted. Participants were allocated into two homogeneous groups (training group, TG, *n* = 40; control group, CG, *n* = 40) by matched features (sex, age, weight and BMI) by the research team. Each group consisted of 20 men and 20 women. Pre-intervention measurements of SaO_2_, VO_2max_ and MWD were taken from each participant 1 week before the training program started in order to establish the baseline. Also, during the training program, the participants were followed up at the 12, 30, and 48^th^ week.

The study has been approved by the Ethics Committee of the Department of Morphological Sciences, School of Medicine, University of Córdoba.

Measurement of Oxygen saturation (SaO_2_). Pulse Oximeter CMS 50DL Finger Pulse Oximeter SaO_2_ was used to measure SaO2. It was measured 1 week prior training; pulse oximeter was utilized to measure SaO_2_ in the right index of each individual. Each individual had to rest for 2 min before the beginning of the measurement. After placing the sensor on the finger, we waited until a reading was displayed on the oximeter, then we waited for another 10–15 s to verify a steady signal, this is followed by recording SaO_2_ and pulse every 10 s. Six observations were recorded and their average was used as the individual’s SaO_2_ as in previous studies [21]. These measurements were taken in a specific cardiopulmonary test, regardless of the training sessions.

Measurement of Maximum Oxygen consumption (VO_2max_). A Cardiopulmonary Exercise Test unit (CPET) Zan 800, a gas analyser of O_2_ and CO_2_ was used for the measurement of VO_2max_. Before conducting the test, the humidity collector was cleaned, the connected tube was checked, and the triple V- valve sensor disinfected and the gas analyser calibrated. The heart rate and blood pressure were recorded in the relaxed sitting position for each subject of the group. The mask was fixed with straps and then the triple tube V was connected to the mask. Initially, metabolic parameters such as oxygen consumption, carbon dioxide production and heart rate at rest were measured every 3 min. These measurements were taken in a specific cardiopulmonary test, regardless of the training sessions.

Maximal Walking Distance (MWD). This data was obtained from the treadmill DKN Run Tech 2.5. MWD is displayed on the screen. MWD data at baseline was recorded from an exercise test, the same test displayed in the first period of training sessions (warming up phase of 5 min on the treadmill, 20 min of active phase, and cooling down phase for a period of 5 min). At the 12th, 30th and 48th week, MWD was recorded from the training session in the corresponding week.

### Intervention

Participants were incorporated in an exercise program of moderate intensity (walking on the treadmill) 3 times weekly for 48 weeks; the first 12 weeks were supervised and the remaining 36 weeks of the program were unsupervised. The duration of complete training sessions was of 30 min the first 3 weeks, 40 min the next 2 weeks, 50 min the next 2 weeks, and 60 min until the end of the program.

The intervention exercise protocol was applied based on the protocol of Naughton [29]. The exercise program consisted of walking on a treadmill with fixed 0 % grade of inclination. The exercise program consists of three phases, which are the followings: a) Warming up phase of 5 min on the treadmill; b) Active phase in which the speed of the treadmill is increased to achieve at least 60 % and not more than 70 % of the maximum heart rate (HR max) according to the protocol of Fletcher and collaborators [22]. The treadmill inclination is fixed at 0 % grade during the whole program, so the intensity of the exercise could be increased or decreased only by changing the speed of the treadmill. The active phase of exercise is 20 min for the first 3 weeks, 30 min for the next 2 weeks, 40 min for the followings 2 weeks and finally for 50 min until the end of the program; c) Cooling down phase for a period of 5 min which is achieved by reducing the speed gradually till reaching zero and until the heart rate returned almost to resting level.

A treadmill DKN Run Tech 2.5 with adjustable speed, inclination and timer, and a large LCD screen with 23 training programs and 3 users’ profiles was used. The screen displays simultaneously walking time and distance, speed, inclination, burned calories and heart rate. The treadmill has front and/or side rails to aid in subject stability. Also, a Pulsometer (HR) p610 Accurex Plus was utilized for monitoring of heart rate.

Participants in the control group were encouraged to walk twice a week during 45 min through enlightening the benefits of moderate physical activity on health, and received standard recommendations for proper health. A telephone follow-up of the adherence to recommended exercise guidelines was conducted weekly.

### Data management and analysis

The collected data was revised, coded, tabulated and introduced to a PC using Statistical package for Social Science (SPSS 19 for Windows). Data was presented and suitable analysis was done according to the type of data obtained for each parameter. Mean and standard deviation were reported to describe quantitative variables.

Prior to the application of the statistical tests to study the effect of the exercise program, initial differences of groups in anthropometric characteristics, age and initial values of the response variables was analysed. Compliance of normality assumption (Shapiro-Wilk test) of these variables in both groups has been verified. When this assumption is violated, we applied the nonparametric Mann-Whitney. If not, t-test for independent samples was applied.

To test the effect of the intervention on SaO_2_, VO_2_ and MWD we applied a Partially Repeated Measures Analysis of Variance. The within factor was the time of measurement and the between factor was the intervention (TG vs CG). Multivariate Test for repeated effects of Time and Time X Group (Pillai’s Trace) was used to evaluate the appropriateness of the application of the multivariate approach; the Mauchly’s test was applied to test the spherical pattern of the covariance matrix. If violated, to test the significance of effects we used repeated testing Huynh-Feldt (HF), which is the least conservative in correcting the effects of the violation of the sphericity assumption. If the significance of the interaction is checked, we studied the trend of time in each group by Repeated Measures Analysis of Variance, multivariate approach. Tukey pairwise comparisons were used to test the differences between time measurements. To reduce sources of variability, the gender of the participants is a blocking factor accounting for treatment variability between both genders. To evaluate the effect size, the partial Eta squared coefficient and the Cohen’s d were used. The level of significance applied was 5 %.

## Results

Prior to the application of the tests to study the effect of the intervention, initial differences of groups by gender in anthropometric characteristics, age and initial values of the response variables was analysed. It is observed that there are no differences between groups in all variables before the intervention (Table 1).

**Table 1:** Comparison of anthropometric values and response variables at baseline

	Females				Males			
	Control Mean (Sd)	Intervention Mean (Sd)	p	d	Control Mean (Sd)	Intervention Mean (Sd)	p	d
	*N* = 20	*N* = 20			*N* = 20	*N* = 20		
Age (Years)	66.1 (2.2)	66.0 (2.3)	.889^a^	.04	64.7 (2.5)	64.3 (2.8)	.594^a^	.15
Weight (Kg)	80.8 (2.0)	81.1 (2.3)	.614^a^	.14	82.2 (1.7)	82.4 (1.8)	.738^b^	.11
Height (Cm)	165.8 (1.4)	165.8 (1.6)	.841^b^	.00	170.4 (1.8)	170.6 (1.8)	.718^b^	.11
BMI (Kg/m^2^)	29.4 (1.0)	29.5 (.9)	.692^a^	.11	28.3 (.5)	28.3 (.7)	.799^b^	.00
SaO2 (%)	97.4 (.2)	97.4 (.2)	.628^a^	.00	96.4 (.2)	96.4 (.2)	.327^b^	.00
VO_2max_ (L/Min)	1.6 (.0)	1.6 (.0)	.602^b^	.00	1.9 (.1)	1.9 (.1)	.678^b^	.00
MWD (M)	660.3 (20.9)	663.6 (21.7)	.621^a^	.15	713.8 (19.8)	715.9 (18.1)	.779^b^	.11

*N* group size, *Sd* standard deviation, *p* significance level, *d* Cohen’s d effect size, *Kg* kilograms, *Cm* centimeters, *m* ^*2*^ squared meters, *L/Min*, liters per minute, *m* meters, *BMI* body mass index, *SaO*_*2*_ oxygen saturation, *VO*_*2max*_ maximum oxygen consumption, *MWD* maximum walking distance

^a^Two independent samples t-test; ^b^Non-parametric two independent samples Mann-Whitney test

The effect of the interaction repeated Time by Group has been found statistically significant, indicating a treatment effect, showing that the values of SaO_2_, VO_2max_ and MWD have a different trend in the intervention group and in the control group. The partial Eta squared coefficient indicates a high effect size of the interaction. Also, there has been a significant main effect for time and group (Table 2). The gender of the participants is a blocking factor accounting for treatment variability between males and females.

**Table 2:** Comparisons of SaO_2_, VO_2max_ and MWD at Baseline, 12, 30 and 48 weeks, and mixed effect model to test the effect Time, Time*Group and Group

Measures	Control	Intervention	Student’s t test			Mixed effect model		
						Within-subjects effects		Between-subjects effects
	Mean (Sd) *N* = 40	Mean (Sd) *N* = 40	t(df)	*p*	Effect size (Cohen’s d) (CI 95 %)	Time F(df) *p* Effect size	Time*Group F(df) *p* Effect size	Group F(df) *p* Effect size
SaO_2_ Bl	96.9 (.5)	96.9 (.5)	.395(78)	0.694	0.00 (−.44;.44)	F(2.5;192.4) = 94.370; *p* <.001; η2 = .554	F(2.5;192.4) = 78.769; *p* <.001; η2 = .509	F(1;76) = 135.937; *p* <.001; η2 = .641
SaO_2_ 12 W	97.0 (.6)	98.4 (.5)	−11.340(78)	<.001	2.33(1.88;2.78)			
SaO_2_ 30 W	97.1 (.5)	97.9 (.8)	−5.273(66.5)^a^	<.001	1.60(1.16;2.04)			
SaO_2_ 48 W	97.0 (.6)	97.7 (1.1)	−3.818(60.0)^a^	<.001	1.17(.73;1.61)			
VO_2max_ Bl	1.8 (.1)	1.8 (.1)	-.009(78)	0.993	0.00 (−.44;.44)	F(2.5;190.1) = 45.422; *p* <.001; η2 = .374	F(2.5;190.1) = 39.061; *p* <.001; η2 = .339	F(1;76) = 85.529; *p* <.001; η2 = .529
VO_2max_ 12 W	1.8 (.1)	2.0 (.1)	−7.057(64.2)^a^	<.001	2.00(1.56;2.44)			
VO_2max_ 30 W	1.8 (.1)	1.9 (.1)	−4.189(78)	<.001	1.00(.56;1.44)			
VO_2max_ 48 W	1.7 (.1)	1.9 (.1)	−5.645(70.0)^a^	<.001	2.00(1.56;2.44)			
MWD Bl	687.0 (33.7)	689.7 (33.0)	-.365(78)	0.716	0.08(−.36;.52)	F(2.5;188.9) = 93.137; *p* <.001; η2 = .551	F(2.5;188.9) = 70.772; *p* <.001; η2 = .482	F(1;76) = 148.211; *p* <.001; η2 = .661
MWD 12 W	697.7 (46.7)	861.7 (57.9)	−13.956(74.6)^a^	<.001	3.74(3.28;4.20)			
MWD 30 W	700.9 (40.6)	796.5 (84.8)	−6.431(56.0)^a^	<.001	2.35(1.90;2.80)			
MWD 48 W	694.8 (31.3)	782.1 (75.5)	−6.753(52.0)^a^	<.001	2.79(2.34;3.24)			

*Sd* standard deviation, *N* group size, *t* t-test for independent samples, *df* degrees of freedom, *p* significance level, *CI 95 %* confidence interval 95 %, *F* Snedecor F statistic, *η* ^*2*^ Partial Eta squared, *SaO*_*2*_ oxygen saturation, *VO*_*2max*_ maximum oxygen consumption, *MWD* maximum walking distance, *Bl* baseline, *12 W* 12 Weeks, *30 W* 30 Weeks, *48 W* 48 Weeks

^a^not homogeneous variances

**Fig. 2:**
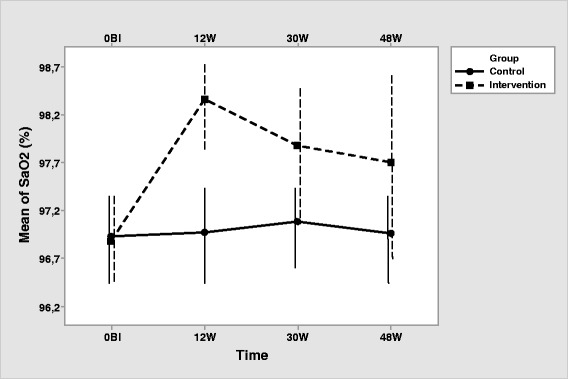
Interaction of group with time for SaO_2_ (Note that the y-axis does not intersect the x-axis at 0). Error bars represent standard deviation

Once the significance of the interaction was checked, we studied the trend of time in each group. In the control group there is a significant time effect for SaO_2_ [F (3, 114) = 5.174, *p* = .002, η^2^ = .120], VO_2max_ [F (3, 114) = 3.376, *p* = .021; η2 = 0.082], and MWD [F (1.98, 75.09) = 7.778, *p* = .001; η^2^ = 0.170]. Tukey pairwise comparisons (Table 3) indicate that SaO_2_ is higher at 30 weeks than in the baseline and 48 weeks (Fig. 2). For VO_2max_, at 30 weeks a greater VO_2max_ than at 48 weeks was found. Regarding MWD, pairwise comparisons (Table 3) indicate that it is lower at baseline than at 12, 30 and 48 weeks. A greater MWD was observed at 30 weeks than at 48 weeks.

**Table 3:** Pairwise comparisons by time at control and intervention groups (positive signs indicate an improvement)

		Control				Intervention			
SaO_2_		MD	Sd	p	d	MD	Sd	p	d
Bl	12 W	0.040	0.290	1.000	0.14	1.487	0.245	<.001	6.07
	30 W	0.155	0.278	.003	0.56	0.995	0.580	<.001	1.72
	48 W	0.030	0.245	1.000	0.12	0.828	0.747	<.001	1.11
12 W	30 W	0.115	0.305	.131	0.38	−0.492	0.523	<.001	0.94
	48 W	−0.010	0.265	1.000	0.04	−0.660	0.733	<.001	0.90
30 W	48 W	−0.125	0.243	0.009	0.51	−0.167	0.621	1.000	0.27
VO_2max_		MD	Sd	*p*	d	MD	Sd	*p*	d
Bl	12 W	0.007	0.054	1.000	0.13	0.200	0.057	<.001	3.51
	30 W	0.014	0.047	.383	0.30	0.130	0.121	<.001	1.07
	48 W	−0.014	0.077	.847	0.18	0.117	0.142	<.001	0.82
12 W	30 W	0.007	0.060	1.000	0.12	−0.070	0.102	<.001	0.69
	48 W	−0.021	0.093	.382	0.23	−0.083	0.121	<.001	0.69
30 W	48 W	−0.028	0.073	.043	0.38	−0.012	0.104	1.000	0.12
MWD		MD	Sd	*p*	d	MD	Sd	*p*	d
Bl	12 W	10.675	23.965	.039	0.45	172.000	37.622	<.001	4.57
	30 W	13.850	16.122	<.001	0.86	106.750	73.880	<.001	1.44
	48 W	7.825	12.786	.002	0.61	92.375	79.845	<.001	1.16
12 W	30 W	3.175	22.020	1.000	0.14	−65.250	67.286	<.001	0.97
	48 W	−2.850	26.405	1.000	0.11	−79.625	90.563	<.001	0.88
30 W	48 W	−6.025	14.961	.009	0.40	−14.375	83.472	1.000	0.17

*MD* mean difference, *Sd* standard deviation, *p* significance level, *d* Cohen’s d effect size, *SaO*_*2*_ oxygen saturation, *VO*_*2max*_ maximum oxygen consumption, *MWD* maximum walking distance, *Bl* baseline, *12 W* 12 Weeks, *30 W* 30 Weeks, *48 W* 48 Weeks

**Fig. 3:**
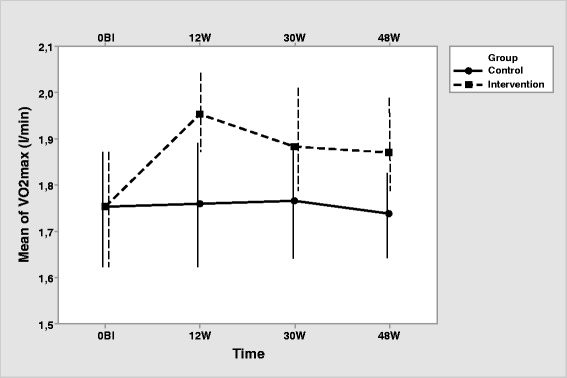
Interaction of group with time for VO_2max_ (Note that the y-axis does not intersect the x-axis at 0). Error bars represent standard deviation

**Fig. 4:**
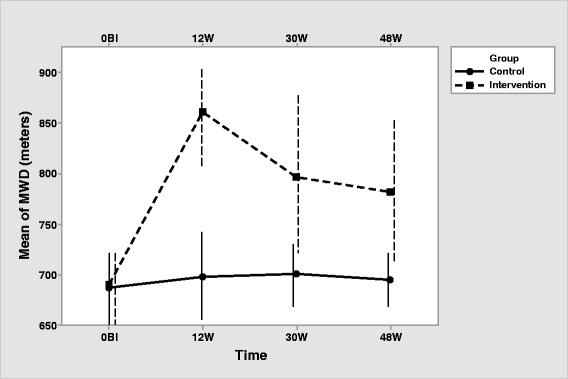
Interaction of group with time for MWD (Note that the y-axis does not intersect the x-axis at 0). Error bars represent standard deviation

The intervention group, as expected, showed a significant effect of the time (HF correction) for SaO_2_ [F (2.25, 85.61) = 55.165, *p* <.001; η2 = 0.592], VO_2max_ [F (2.25, 85.61) = 55.165, *p* <.001; η^2^ = 0.592], and MWD [F (2.38, 90.58) = 87.806, *p* <.001; η^2^ = 0.698]. Pairwise comparisons (Table 3) show an increase in SaO_2_, VO_2max_ and MWD from baseline to 12, 30 and 48 weeks. Highest SaO_2_, VO_2max_ and MWD is observed at 12 weeks, descending at 30 and 48 weeks, showing that supervised exercise produces better outcomes that unsupervised exercise (Figs. 2, 3 and 4). At 30 and 48 weeks, SaO_2_, VO_2max_ and MWD are similar, showing maintained intervention effects at the unsupervised period. All participants in both, intervention and control group, completed the corresponding exercise protocol, thus specific measurements of compliance were not registered.

Concerning the differences between groups at each time, there was no difference in SaO_2_, VO_2max_ and MWD between the two groups in the pretest. All three measurements are higher in the intervention group at 12, 30 and 48 weeks (Table 2, Figs. 2, 3 and 4).

## Discussion

The objective of the current intervention study was to evaluate the therapeutic efficacy of treadmill exercises on SaO_2_, VO_2max_ and MWD in elderly healthy subjects. The results of this study show: a) that treadmill exercise produces a beneficial effect on these parameters in healthy elderly people; and b) that supervised exercises has better results than unsupervised exercise.

### Intervention effects on pulmonary outputs (SaO_2_, VO_2max_ and MWD)

We investigated the effect of controlled exercise (first aspect) on a treadmill (second aspect) in some parameters of routine clinical use. We meant to evaluate how programmed physical exercise can affect the physiological status of the elderly, improving their cardiopulmonary health, without including their quality of life. This was achieved by monitoring the physical activity and using objective data.

After the first 12 weeks of training (the controlled short-term program) there was a highly significant increase of the three variables of the study in the intervention group comparing it before and after the exercise program as well as with the control group. The physiological explanation is obvious because dealing with healthy elderly people who have made a controlled physical activity which dramatically improved the three variables of the study and the results have been highly positive. The control group also achieved an improvement in pulmonary outputs, but smaller than that from the intervention group. Thus, greater results in the intervention group could be related to the fact that both exercise programs were not dose-match, which, in turn, could lead to better achievement in the greater dose group. But this is not perceived as a limitation since we aim to compare a well-planned exercise program with standard recommendations for moderate physical activity in elderly people. Also, a highly significant improvement appears in the data at 30 and 48 weeks in the group that has complied with the unsupervised long term program, as compared with the control group.

The physical and physiological benefits achieved and the results showed match those given by Figoni et al. [30], whose relatively recent study found that after a training exercise program for 3 months, also with treadmills, and elderly people (mean age 69 years), an improvement was shown in both walking distance, and in arterial oxygen saturation. Also, Kalapotharakos, [31] following an aerobic training program, showed that there was an increase from 6.6 to 30 % VO_2max_ in seniors. And, taking into account the study published by Wong et al. [32], every minute of normal gait increased per day leads to an increase in VO_2max_ by 0.096 ml/kg/min. Even years ago, Shephard [33], showed that prolonged aerobic physical activity can reverse the decline in VO_2max_ recovering and, consequently, the corresponding metabolic equivalent can increase in young moderately active subjects from 12 METs to18-24 METs by performing aerobic training such as distance running [22].

Meanwhile, Huang et al. [34] found that there was no change in SaO_2_ in a group of 16 women, whose ages ranged from 80 to 93 years, who participated in an exercise training program for 4 months. In the article it is unclear whether the lack of samples gave no significant statistical values or other factors influenced the study such as the advanced age of the group or even the type of training intervention.

These findings highlight that participation of older adults in aerobic exercise programs significantly improves cardiopulmonary response [31]. Treadmill walking may bring important health benefits in term of improvement in physical performance, fitness and its implications for the prevention of physical disability in older adults. This also reinforces the theory that low-to moderate intensity activities may improve cardio-respiratory fitness [32].

### Supervised vs Unsupervised exercise effects

Bendermacher et al. [23], demonstrated the existence of significant differences and obvious clinical differences, when after a supervised exercise regimen, subjects improved distance walked on the treadmill compared with the unsupervised training for 3 months, still, the overall effect size of 0.59 (95 % confidence interval 0.31 to 0.85), the improvement resulted in a difference of about 150 m. In addition, the improved results in increasing the walking ability are maintained after the end of the program. As in our study, this study clearly shows greater improvement in cardiopulmonary outputs in response to supervised exercise program in healthy elderly. We have found that a supervised period of exercise program is related to better results in SaO_2_, VO_2max_ and MWD values.

A previous study found a positive relationship between compliance and feedback [35]. Patients whose compliance were monitored and fed back on its developments and progress had more compliance than patients without supervision. This coincides substantially with the results exposed. Also, a few years ago, it was shown that compliance is reduced from 59 % in a supervised program to 29 % after 6 months of unsupervised exercise, concluding that patient compliance with physical therapy is unsatisfactory but monitoring improves adherence [36]. In our study, compliance, measured as the proportion of participants completing the exercise protocol in both the intervention and control group, was 100 %. This is a significant strength of the study, since achievement of adherence to an exercise program for nearly 1 year is a great achievement, showing that prescription of moderate exercise is well accepted in healthy elderly people. However, results showed that the supervised period of the intervention achieved greater improvement in pulmonary outputs, which could be related to motivation or compliance with instructions and encouragement from monitoring.

### Limitations

Limitations such as the small sample size could affect the validity of the results [37]. Our study included only 80 elderly healthy subjects. It was too difficult to identify more subjects between 60 and 70 years old without any chronic diseases who accepted to participate in a follow up study for nearly 1 year.

Another possible limitation was each participant’s psychological status at the time of exercise and his or her compliance to the supervised program. Motivation is an essential construct related to engagement in sustained physical activities [38], so the lack of motivation could lead to diminished compliance of the exercise program instructions. Although these limitations could not be avoided, our findings strongly suggest that exercise training programmes on a treadmill (short or long-term) can improve VO_2max_, SaO_2_ and maximum walking distance in elderly healthy subjects and the health benefits of the 3 parameters are closely related to the fitness of the subjects to the exercise program parameters, as showed in the supervised period of the intervention.

The purpose of this study was to investigate the effect of exercise and activity in older people in the Republic of Egypt considering that while in other nations and cultures these types of studies are quite frequent and started to be applied, in Egypt it is not so common because the average life expectancy and general health condition of the population is much lower than in Europe, and the practice of physical activity in old age is far from being an established plan. Consequently, it was difficult precisely at this time where the activity took place to do similar studies, the results had to be contrasted and their explanations had to be found.

## Conclusions

The results of this study suggest that it is highly recommended for healthy older people to perform moderate exercise, specifically walking several days a week. Indicators of pulmonary capacity of the elderly (SaO_2_, VO_2max_ and MWD) have improved from the practice of moderate exercise. Primary care physicians should consider this and other studies and strongly recommend older people living habits that include moderate physical activity in the ordinary way. Our findings were consistent and coherent, strongly indicating the external validity of the study.

## Abbreviations

12 W: 12 weeks
30 W: 30 weeks
48 W: 48 weeks
Bl: baseline
BMI: body mass index
CG: control group
CI95%: confidence interval 95 %
d: Cohen’s effect size
df: degrees of freedom
HF: Huynh-Feldt test
MWD: maximum walking distance
N: group size
NRCT: non-randomized controlled trial
p: significance level
t: t-test for independent samples
TG: training group
SaO2: arterial blood oxygenation
Sd: standard deviation
VO2max: maximum oxygen consumption
η^2^: partial eta-squared
